# PFOA-Induced Ovotoxicity Differs Between Lean and Obese Mice With Impacts on Ovarian Reproductive and DNA Damage Sensing and Repair Proteins

**DOI:** 10.1093/toxsci/kfac104

**Published:** 2022-10-10

**Authors:** Maria Estefanía González-Alvarez, Andrew Severin, Maryam Sayadi, Aileen F Keating

**Affiliations:** Department of Animal Science and Interdepartmental Toxicology Graduate Program, Iowa State University, Ames, Iowa 50011, USA; Department of Animal Science and Interdepartmental Toxicology Graduate Program, Iowa State University, Ames, Iowa 50011, USA; Department of Animal Science and Interdepartmental Toxicology Graduate Program, Iowa State University, Ames, Iowa 50011, USA; Department of Animal Science and Interdepartmental Toxicology Graduate Program, Iowa State University, Ames, Iowa 50011, USA

**Keywords:** PFOA, obesity, ovary, DNA damage sensing and repair, reproduction, ovarian proteome

## Abstract

Perfluorooctanoic acid (PFOA) is an environmentally persistent perfluoroalkyl substance that is widely used in consumer products. Exposure to PFOA is associated with reproductive and developmental effects including endocrine disruption, delayed puberty in girls, and decreased fetal growth. In the United States, obesity affects 40% of women and 20% of girls, with higher rates in minority females. Obesity causes infertility, poor oocyte quality, miscarriage, and offspring defects. This study proposed that PFOA exposure would impact estrous cyclicity, ovarian steroid hormones, and the ovarian proteome and further hypothesized that obesity would impact PFOA-induced ovotoxicity. Female wild type (KK.Cg-a/a; lean) or KK.Cg-Ay/J mice (obese) received saline (CT) or PFOA (2.5 mg/kg) *per os* for 15 days beginning at 7 weeks of age. There were no effects on food intake, body weight, estrous cyclicity, serum progesterone, and heart, spleen, kidney, or uterus weight (*p *>* *.05). Ovary weight was decreased (*p *<* *.05) by PFOA exposure relative to vehicle control-treated mice in lean but not obese mice. Liquid chromatography-tandem mass spectrometry was performed on isolated ovarian protein and PFOA exposure altered the ovarian abundance of proteins involved in DNA damage sensing and repair pathways and reproduction pathways (*p *<* *.05) differentially in lean and obese mice. The data suggest that PFOA exposure alters ovary weight and differentially targets ovarian proteins in lean and obese females in ways that might reduce female fecundity.

The female reproductive system performs important functions that support fertilization and pregnancy (Hoyer, 2010). The ovary has 2 major roles: production and secretion of 17β-estradiol (E_2_) and progesterone (P_4_), and development of oocytes (Hirshfield, 1991). Females are born with a finite number of oocytes (Hirshfield, 1991) that are ovulated or undergo atresia (Fortune *et al.*, 2000). However, several factors including exposure to xenobiotics (Hoyer, 2005; Keating and Hoyer, 2009; Mattison, 1985) can affect proper ovarian functioning causing follicle loss (Keating *et al.*, 2009; Klenov *et al.*, 2021; Madden *et al.*, 2014), DNA damage (Clark and Keating, 2020; Ganesan and Keating, 2016; Winship *et al.*, 2020), and endocrine disruption (Hoyer, 2005) leading to temporary or permanent infertility (Hoyer and Keating, 2014).

The prevalence of obesity among adults in the United States is approximately 42% (Hales *et al.*, 2020) with approximately 40% of women over 20 years and 20% of girls being obese (Hales *et al.*, 2017, 2020). Minority women are affected by obesity at higher rates (Hales *et al.*, 2020). Obesity is now commonplace in developed countries and has increased also in developing countries (OECD, 2017; Rössner, 2002; WHO, 2021). Obesity can lead to chronic diseases including cardiovascular disease (Rössner, 2002), type 2 diabetes (Rössner, 2002), dyslipidemia (Rössner, 2002), and hypertension (Rössner, 2002). In women, obesity has negative reproductive effects (Aune *et al.*, 2014; Chu *et al.*, 2007; Grodstein *et al.*, 1994; Jungheim *et al.*, 2010; McDonald *et al.*, 2010; Pasquali *et al.*, 2003; Pasquali and Casimirri, 1993; Rich-Edwards *et al.*, 1994; Smith *et al.*, 2007; Stothard *et al.*, 2009; Watkins *et al.*, 2003) including decreased fecundity (Grodstein *et al.*, 1994; Rich-Edwards *et al.*, 1994), poor oocyte quality (Jungheim *et al.*, 2010), gestational diabetes (Chu *et al.*, 2007), increased risk of birth defects (Stothard *et al.*, 2009; Watkins *et al.*, 2003), premature (McDonald *et al.*, 2010; Smith *et al.*, 2007), and still births (Aune *et al.*, 2014) and is associated with polycystic ovarian syndrome (Pasquali and Casimirri, 1993). Obesity induces oxidative DNA damage and oxidative stress (Ganesan *et al.*, 2017), induces basal DNA damage (Ganesan *et al.*, 2014, 2017), alters phosphatidylinositol-3 kinase (PI3K) signaling (Nteeba *et al.*, 2013, 2017), impairs the response of ovarian chemical metabolism proteins (Nteeba *et al.*, 2014a, 2017), and depletes primordial follicles (Ganesan *et al.*, 2017; Nteeba *et al.*, 2014a,b). These findings suggest that the ovaries of obese females may be more sensitive to reproductive toxicants.

When DNA damage is detected, several mechanisms involved in the DNA damage repair (DDR) response are activated to prevent the DNA damage to be passed to daughter cells (Friedberg, 2003; Zhou and Zheng, 2013). Mammalian DNA repair pathways include base-excision repair (BER), homologous recombination (HR), nucleotide-excision repair (NER), and nonhomologous end joining (NHEJ) (Hoeijmakers, 2001). Single-strand DNA breaks (SSBs) are repaired by NER and BER mechanisms (Hoeijmakers, 2001). Transcription and normal replication can be affected mostly by exogenous sources that lead to helix-distorting lesions that are targeted and repaired by NER mechanisms (Hoeijmakers, 2001). Methylation, reactive oxygen species, hydroxylation, deamination, and chemical alterations of bases endogenously induced that alter transcription and replication are repaired by BER pathways (Hoeijmakers, 2001). Double-strand breaks (DSBs) are repaired by HR and NHEJ pathways (Hoeijmakers, 2001). When DNA replication is occurring to align the breaks in S and G2 phases, HR provides a second copy of the sequence, thus repairing the damage (Hoeijmakers, 2001). If a second copy is not available, NHEJ pathway intervenes on the G1 phase of the cell cycle to repair the damage (Hoeijmakers, 2001). DNA damage has been associated with aging (Finkel and Holbrook, 2000), genetic disorders (Finkel and Holbrook, 2000), and cancer (Hoeijmakers, 2001).

Per- and poly-fluoroalkylated substances (PFAS) are characterized by having strong bonds between carbon and fluorine groups (EFSA, 2008; Lau, 2012; USEPA, 2017) which confer both thermal and chemical stability (Bell *et al.*, 2021) making PFAS resistant to degradation (EFSA, 2008) and persistent in the environment (Lau, 2012; ATSDR, 2021). Humans are exposed though ingestion, inhalation, and through dermal exposure (Post *et al.*, 2012). Perfluorooctanoic acid (PFOA) is a fluorinated organic acid PFAS member found in consumer goods including nonstick cookware, water and stain-resistant carpet and fabric coatings, floor polish, fire-fighting foam, lubricants, and food packaging (EFSA, 2008; Post *et al.*, 2012). Exposure to PFOA has been associated with hepatoxicity (EFSA, 2008; Post *et al.*, 2012; ATSDR, 2021), carcinogenicity (Chang *et al.*, 2014), developmental toxicity (EFSA, 2008; Post *et al.*, 2012; ATSDR, 2021), and reproductive toxicity (EFSA, 2008; Post *et al.*, 2012; ATSDR, 2021). In females, exposure to PFOA has been linked to delayed puberty in girls (Lopez-Espinosa *et al.*, 2011), early menopause (Knox *et al.*, 2011), endocrine disruption (Jensen and Leffers, 2008; Knox *et al.*, 2011; Yang *et al.*, 2022), reduced fertility (Fei *et al.*, 2009; Vélez *et al.*, 2015), and premature ovarian insufficiency (Zhang *et al.*, 2018). It has been detected in follicular fluid of women (Heffernan *et al.*, 2018) and is associated with decreased fetal growth (Gyllenhammar *et al.*, 2018) and reduced childhood growth (Andersen *et al.*, 2010). In mice, delayed puberty (Lau, 2012), changes in the female reproductive tract (Dixon *et al.*, 2012), altered levels of steroid hormones (Chen *et al.*, 2017; Lau, 2012), follicle loss (Yang *et al.*, 2022), and reduced number and size of corpora lutea (Chen *et al.*, 2017) have been observed due to exposure to PFOA.

Despite evidence indicating that PFOA causes reproductive toxicity, the mechanisms are not well understood. The purpose of this study was to investigate mechanisms of PFOA-induced ovarian toxicity including alterations to the levels of E_2_ and P_4_, and changes to the ovarian abundance of proteins involved in reproduction, and DNA damage sensing and repair. Additionally, an obese group of mice were included to ascertain if obesity would impact the ovarian impacts of PFOA exposure.

## MATERIALS AND METHODS

###  

####  

##### Reagents

PFOA (CAS no. 335-67-1), phosphate-buffered saline (PBS), tris-HCl, tris-buffered saline (TBS), and nonfat dry milk were purchased from Sigma-Aldrich Inc. (St Louis, Missouri). Pierce bicinchoninic acid assay (BCA) Protein Assay Kit was obtained from Thermo Fisher Scientific (Rockford, Illinois). Glycine and Tween 20 were obtained from Fisher Bioreagents (Fair Lawn, New Jersey). E_2_ and P_4_ ELISA kits were purchased from DRG International, Inc. (Springfield, New Jersey).

##### Animal exposure

The Iowa State University Institutional Animal Care and Use Committee approved all the animal protocols for the study. Female wild-type normal non-agouti KK.Cg-a/a mice, designated lean hereafter (*n* = 19), and agouti lethal yellow KK.Cg-Ay/J mice, designated obese (*n* = 20), were obtained from Jackson Laboratories (Bar Harbor, Maine) at 5 weeks of age. Under identical controlled conditions: a light cycle of 12 h light/12 h darkness, temperature between 21°C and 22°C, and 20%–30% humidity, mice were housed in Innovive cages with 2 or 3 animals per cage. The mice were fed 2014 Teklad Global 14% Protein Rodent Diet and water *ad libitum*. Body weight gain and food intake were monitored twice per week. Food intake was calculated as food disappearance per cage/number of mice per cage. After 2 weeks of acclimation, at 7 weeks of age, mice were dosed with either saline solution as vehicle control (CT) or PFOA (2.5 mg/kg; 2.5 ppm) *per os* from a pipette tip for 15 days. There were 4 treatment groups—lean mice treated with vehicle control (LC); lean mice exposed to PFOA (LP); obese mice treated with vehicle control (OC), and obese mice exposed to PFOA (OP). The dose chosen was intermediate for the lowest observable adverse effect level (LOAEL) noted for PFOA in the testis (5 mg/kg; Li *et al.*, 2011) and the liver (1 mg/kg; Loveless *et al.*, 2008). The age of 7 weeks was chosen to avoid the decline in primordial and primary follicles in the obese mice which occurs from 12 weeks onwards (Ganesan *et al.*, 2017; Nteeba *et al.*, 2014a,b).

##### Monitoring of the estrous cycle

For 14 days, estrous cyclicity was monitored by performing vaginal cytology. The vagina was gently flushed with saline 3–5 times, using a sterile pipette tip (Caligioni, 2009). The final flush was collected and observed under the microscope (Caligioni, 2009). In the proestrus stage, most cells are nucleated epithelial, but some cornified and leucocytes are present (Byers *et al.*, 2012; Caligioni, 2009). In the estrus stage, large cornified cells with irregular shape and no visible nucleus appear (Byers *et al.*, 2012; Caligioni, 2009). During the metestrus stage, predominantly leukocytes appear, but some cornified epithelial and less nucleated epithelial cells are also apparent (Byers *et al.*, 2012; Caligioni, 2009). Mostly polymorphonuclear leukocytes appear during the diestrus stage with few nucleated epithelial cells (Byers *et al.*, 2012; Caligioni, 2009). Because of the similarity in cytology for the metestrus and diestrus stage, these 2 stages were combined. Statistical analysis was performed on the raw data and for the purpose of visualization, the percentage of time spent at each stage was calculated by the number of days per stage/14 days × 100.

##### Tissue collection

Mice were euthanized by CO_2_ asphyxiation followed by cervical dislocation on the second day of diestrus stage of the estrous cycle and tissues were collected. Cardiac puncture was performed to collect blood samples. The heart, spleen, kidneys, uterus, and ovaries were collected and weighed after trimming each of any excess fat. One ovary was flash frozen in liquid nitrogen and stored at −80°C for protein analysis.

##### Serum E_2_ and P_4_ hormone level quantification

Blood samples were centrifuged for 15 min at 10 621 rcf and 4°C, followed by discarding blood cells. Serum E_2_ (DRG Estradiol ELISA; EIA-2693; CV = 35.2) and P_4_ (DRG Progesterone ELISA; EIA-1561; CV = 27.8) were quantified by ELISA following the manufacturer’s instructions with 2 technical replicates per sample. For E_2_ quantification, there was sufficient serum for 39 samples (LC = 10; LP = 9; OC = 10; OP = 10) and adequate serum for measurement of 36 samples for P_4_ (LC = 10; LP = 9; OC = 9; OP = 8). Several samples were below the detectable range of the E_2_ assay (LC = 7; LP = 5; OC = 3; OP = 4) and for those samples, the limit of detection of the assay (10.6 pg/ml) was divided by √2 (Ogden, 2010) for inclusion in the statistical analysis. Additionally, E_2_ assay results were analyzed to include only samples that were within the detectable range of the assay: LC = 3; LP = 4; OC = 7; OP = 6. All samples were within the analytical range of the P_4_ assay.

##### Protein isolation

Total ovarian protein was isolated by homogenizing ovaries in lysis buffer (50 mM Tris-HCl and 1 mM EDTA; pH approximately 8.5). Samples (LC = 10; LP = 9; OC = 10; OP = 10) were centrifuged twice for 15 min at 10 621 rcf and the supernatant collected each time. A BCA assay was performed to measure protein concentration. Absorbance values were detected at 560 nm by an Eon Microplate Spectrophotometer (Bio-Tek Instruments Inc., Winooski, Vermont).

##### LC-MS/MS proteome analysis

Liquid chromatography-tandem mass spectrometry (LC-MS/MS) analysis was performed for protein separation and identification as described previously (Clark *et al.*, 2019). Briefly, total protein samples (LC = 5; LP = 5; OC = 5; OP = 5) were digested with trypsin/Lys-C for 16 h, dried down and reconstituted in buffer A (47.5 µl, 0.1% formic acid/water). An internal control, Peptide Retention Time Calibration (PRTC), was spiked into each sample. Protein samples and PRTC were injected onto a liquid chromatography column (Agilent Zorbax SB-C18, 0.5 mm × 150 mm, 5 µm) to be separated and analyzed with a mass spectrometer. Fragmentation patterns and intact results were compared with theoretical fragmentation patterns from MASCOT or Sequest HT to identify peptides.

##### Statistical analysis

Statistical analyses were performed on all endpoints with the exception of the LC-MS/MS data using GraphPad Prism 8.4.1 software. Two-way analysis of variance (2-way ANOVA) was performed to compare 2 independent variables (body composition and PFOA exposure and any interaction) using Tukey’s multiple comparison test. A *p* value ≤ .05 was defined as a statistically different result between treatments.

For the LC-MS/MS analysis, Metaboanalyst 3.0 (Xia *et al.*, 2015; Xia and Wishart, 2016) was used for data analysis. Upon finding data integrity to be satisfactory (no peptide with more than 50% missing replicates, positive values for the area), missing value imputation was performed using a singular value decomposition method. Filtering, based on interquartile range, was performed to remove values that are unlikely to be of use when modeling the data, followed by generalized log transformation before data analysis. The relevant control and treatment samples were compared using a Student *t* test. Differences between groups were assessed by the Mann-Whitney rank sum test. All *p* values were 2 sided. To adjust for multiple comparisons, Bonferroni correction was applied and only *p* values less than .05 were considered significant. The PCA analysis was performed using the *prcomp* package and pairwise score plots providing an overview of the various separation patterns among the most significant components were accessed. The PLS regression was then performed using the *plsr* function provided by R *pls* package. The classification and cross-validation were also performed using the *caret* package. The Uniprot protein identifiers were used to retrieve biological pathway association of the proteins using DAVID 6.8 software.

## RESULTS

###  

#### Impact of PFOA Exposure on Food Intake and Body Weight Gain in Lean and Obese Mice

**Figure 1.**
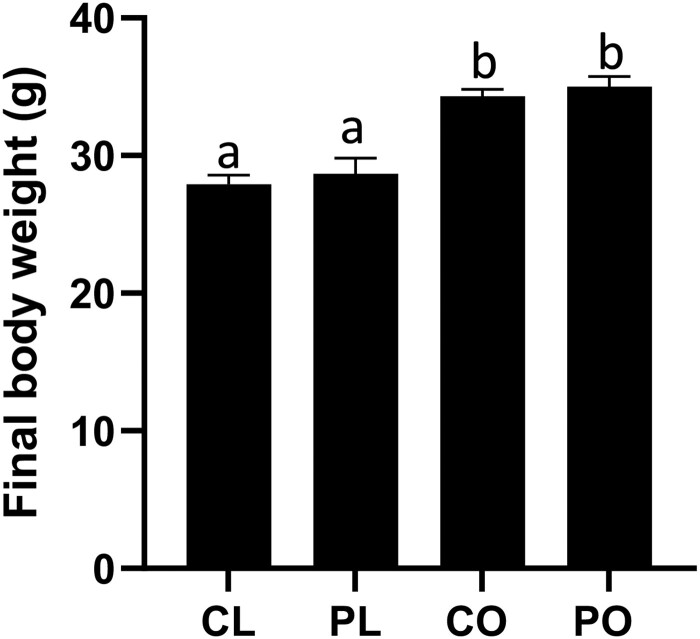
Effects of PFOA exposure on body weight in lean and obese mice. Mice were weighed prior to euthanasia. Bars represent body weight (g) ± SEM. Superscript letters indicate significant differences; *p *<* *.05. LC, lean control-treated mice; LP, lean PFOA-exposed mice; OC, obese control-treated mice; OP, obese PFOA-exposed mice. *n*: LC = 10; LP = 9; OC = 10; OP = 10.

PFOA exposure did not affect food intake (*p *>* *.05) in lean or obese mice, however, as expected, mean food intake was higher in the obese compared with lean mice (LC = 62.45 ± 1.1 g; LP = 65.0 ± 1.7 g; OC = 82.0 ± 2.5 g; OP = 79.6 ± 3.1 g). At euthanasia, the obese mice were approximately 24% heavier (*p *<* *.05) than their lean counterparts (Figure 1). Body weight was not affected (*p *>* *.05) by PFOA exposure in lean or obese mice (Figure 1; treatment effect *p *=* *.36; obesity effect *p* ≤ .0001; interaction effect *p *=* *.97).

#### Effects of PFOA Exposure on Organ Weight in Lean and Obese Mice

**Figure 2.**
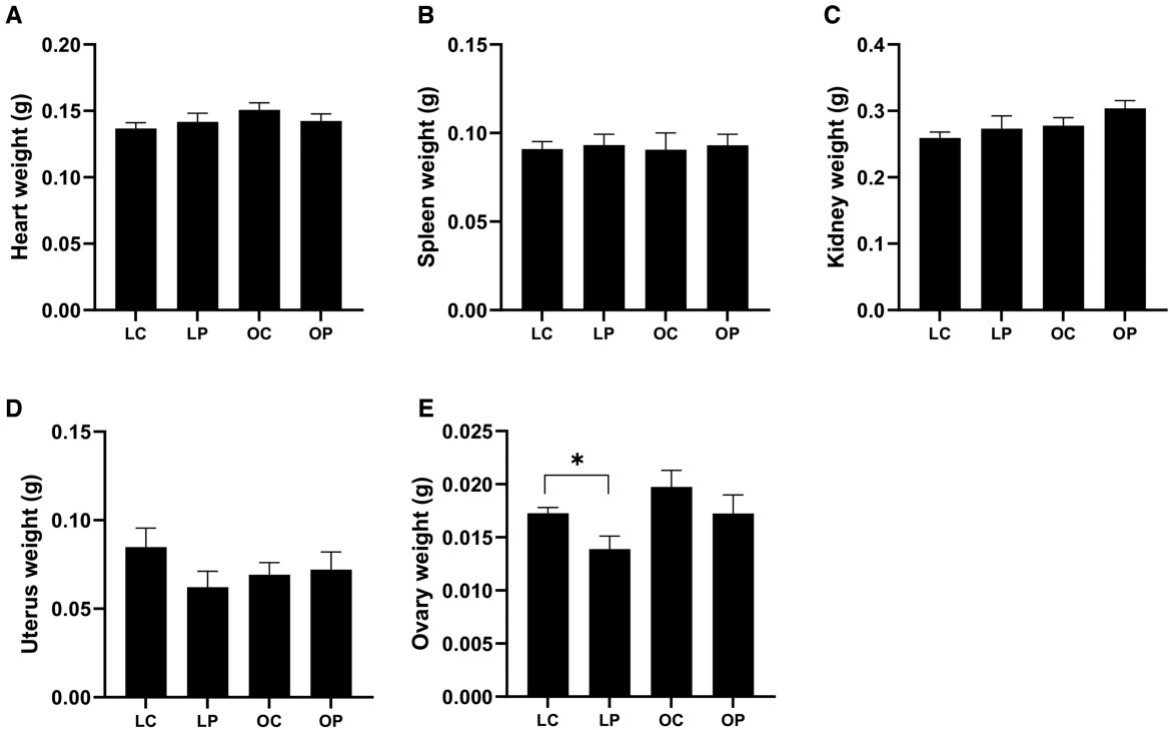
Effect of PFOA exposure on organ weight in lean and obese mice. After euthanasia, (A) heart, (B) spleen, (C) kidney, (D) uterus, and (E) ovary were collected and weighed (g). Bars represent mean weight ± SEM. Asterisks indicate statistical difference; *p *<* *.05. LC, lean control-treated mice; LP, lean PFOA-exposed mice; OC, obese control-treated mice; OP, obese PFOA-exposed mice. *n*: LC = 10; LP = 9; OC = 10; OP = 10.

There was no effect of PFOA or obesity (*p *>* *.05) on (A) heart (treatment effect *p *=* *.76; obesity effect *p *=* *.18; interaction effect *p *=* *.22; Figure 2A), (B) spleen (treatment effect *p *=* *.72; obesity effect *p *=* *.97; interaction effect *p *=* *.98; Figure 2B), (C) kidney (treatment effect *p *=* *.14; obesity effect *p *=* *.07; interaction effect *p *=* *.65; Figure 2C), and (D) uterus (treatment effect *p *=* *.29; obesity effect *p *=* *.75; interaction effect *p *=* *.18; Figure 2D). Ovary weight was decreased (treatment effect *p *=* *.038; obesity effect *p *=* *.038; interaction effect *p *=* *.76; Figure 2E) due to PFOA exposure in lean, but not in obese mice (LC = 0.017 g ± 0.0005; LP = 0.014 g ± 0.001; OC = 0.02 g ± 0.001; OP = 0.017 g ± 0.001), relative to their respective vehicle control-treated counterparts.

#### Alterations to Ovarian Steroid Hormone Level and Estrous Cyclicity due to PFOA Exposure in Lean and Obese Mice

**Figure 3.**
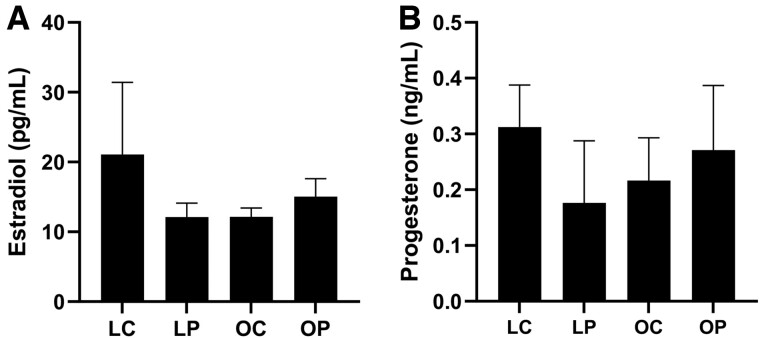
Ovarian steroid hormone impact of PFOA exposure in lean and obese mice. Circulating E_2_ and progesterone were measured by ELISA. LC, lean control-treated mice; LP, lean PFOA-exposed mice; OC, obese control-treated mice; OP, obese PFOA-exposed mice. Bars represent mean concentration ± SEM. E_2_*n*: LC = 10; LP = 9; OC = 10; OP = 10. P_4_*n*: LC = 10; LP = 9; OC = 9; OP = 8.

**Figure 4.**
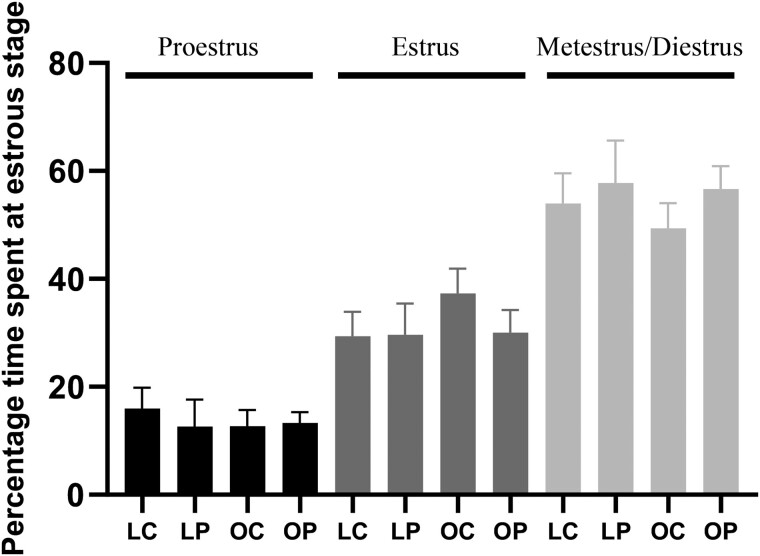
Estrous cyclicity impact of PFOA exposure in lean and obese mice. The number of days at each stage of the estrous cycle were calculated over a 14-day period and presented as a percentage. Bars represent percentage of day at proestrus (black bars), estrus (dark gray bars), and metestrus + diestrus (light gray bars) ± SEM. Asterisk indicate differences between treatments; *p *<* *.05. LC, lean control-treated mice; LP, lean PFOA-exposed mice; OC, obese control-treated mice; OP, obese PFOA-exposed mice. *n*: LC = 10; LP = 9; OC = 10; OP = 10.

Detectable E_2_ levels were present in 37% of lean and 65% of obese mice regardless of PFOA exposure with a high level of variation in the lean vehicle control-treated mice. Circulating E_2_ was increased (*p *<* *.05) in obese but not lean mice by PFOA exposure when samples below the limit of detection were omitted (LC = 52.8 ± 29.7; LP = 17.9 ± 2.1; OC = 14.2 ± 1.1; OP = 20.1 ± 2.6). Inclusion of samples that fell below the limit of detection of the assay by dividing the lower detection limit by √2 indicated no effect of PFOA exposure on circulating E_2_ (treatment effect *p *=* *.59; obesity effect *p *=* *.60; interaction effect *p *=* *.29; Figure 3A) in lean or obese mice. The level of circulating P_4_ was not affected (treatment effect *p *=* *.67; obesity effect *p *=* *.99; interaction effect *p *=* *.32) by obesity or PFOA exposure in any of the groups (Figure 3B). Using ELISA as a quantification method, the level of circulating E_2_ was in the published range for mice in the diestrus phase of the estrous cycle, however P_4_ levels were lower than previously noted (Zenclussen, 2014). There was no effect of obesity, PFOA exposure, or additive impact of obesity and PFOA on the time spent at proestrus (treatment effect *p *=* *.70; obesity effect *p *=* *.72; interaction effect *p *=* *.57), estrus (treatment effect *p *=* *.47; obesity effect *p *=* *.39; interaction effect *p *=* *.43), and metestrus + diestrus (treatment effect *p *=* *.33; obesity effect *p *=* *.61; interaction effect *p *=* *.75; Figure 4).

#### Effects of PFOA Exposure and Obesity on the Global Ovarian Proteome

**Figure 5.**
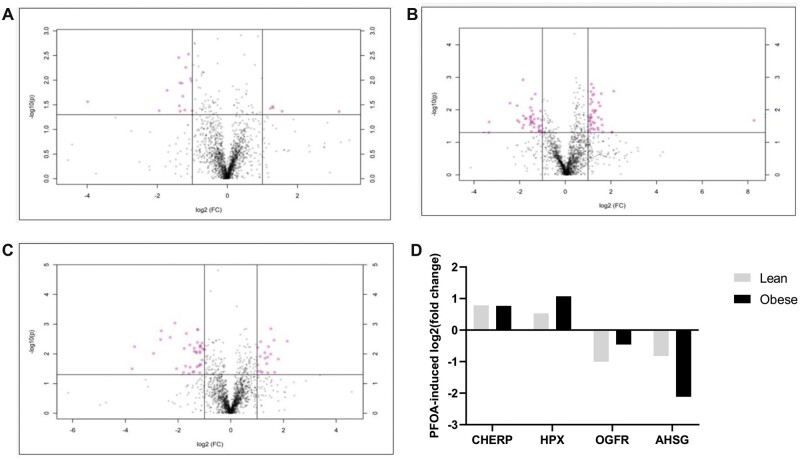
Impact of PFOA on the ovarian proteome in lean and obese mice. Total ovarian protein homogenates were analyzed by LC-MS/MS and bioinformatic comparison performed between peptides identified in (A) LC versus LP, (B) OC versus OP, and (C) LC versus OC. Dots above the solid horizontal line indicate increased (upper right corner) or decreased (upper left corner) proteins; *n* = 5/treatment; *p *<* *.05. D, The log2(fold-change) of proteins increased (CHERP and HPX) and decreased (OGFR and AHSG) by PFOA in both lean (gray bar) and obese (black bar) mice.

In lean mice, exposure to PFOA altered the abundance of 98 proteins (*p *<* *.05). Of these, the level of 36 were increased and 62 were decreased (Figure 5A). The biological or molecular functions of 16 (4 increased and 12 decreased) proteins are involved in DNA damage and repair (Table 1), and 7 (2 upregulated and 5 downregulated) in reproduction (Table 2).

**Table 1.** Altered Ovarian Abundance of Proteins Involved in DNA Damage Sensing and Repair in Lean PFOA-Exposed Relative to Lean Vehicle Control-Treated Mice

UniProtID	Protein Name	Protein Abbreviation	*p* Value	Log2(Fold Change)
Q64522	H2A clustered histone 21	H2AC21	.036	1.31
P68433	H3 clustered histone 1	H3C1	.037	1.23
Q08943	Structure-specific recognition protein 1	SSRP1	.036	0.73
P43274	H1.4 linker histone, cluster member	H1-4	.016	0.34
P07901	Heat shock protein 90 alpha family class A member 1	HSP90AA1	.001	−0.28
P60335	Poly(rC)-binding protein 1	PCBP1	.004	−0.28
Q921I1	Transferrin	TF	.029	−0.38
O70456	Stratifin	SFN	.026	−0.42
Q8VDP4	Cell cycle and apoptosis regulator 2	CCAR2	.042	−0.46
Q3TCH7	Cullin 4A	CUL4A	.037	−0.6
P34884	Macrophage migration inhibitory factor	MIF	.044	−0.6
P26350	Prothymosin alpha	PTMA	.007	−0.70
Q923G2	RNA polymerase I, II, and III subunit H	POLR2H	.040	−0.71
P53996	CCHC-type zinc finger nucleic acid-binding protein	CNBP	.003	−0.83
E9QAM5	Helicase with zinc finger 2	HELZ2	.005	−0.96
Q3TKT4	SWI/SNF related, matrix associated, actin-dependent regulator of chromatin, subfamily a, member 4	SMARCA4	.040	−1.21

UniprotID refers to protein identifiers on Uniprot.

Protein abbreviation is the abbreviation for each altered protein.

Log2 refers to the log2 fold change in PFOA exposed relative to vehicle control-treated lean mice.

**Table 2.** Altered Ovarian Abundance of Proteins Involved in Reproduction in Lean PFOA-Exposed Relative to Lean Vehicle Control-Treated Mice

UniProtID	Protein Name	Protein Abbreviation	*p* Value	Log2(Fold Change)
P70274	Selenoprotein P	SELENOP	.034	1.29
P26361	CF transmembrane conductance regulator	CFTR	.047	0.84
P34884	Macrophage migration inhibitory factor	MIF	.044	−0.62
Q9CYR6	Phosphoglucomutase 3	PGM3	.032	−0.67
Q9JKV1	ADRM1 26S proteasome ubiquitin receptor	ADRM1	.038	−0.78
P29699	Alpha-2-HS glycoprotein	AHSG	.014	−0.82
Q3TKT4	SWI/SNF related, matrix associated, actin-dependent regulator of chromatin, subfamily a, member 4	SMARCA4	.040	−1.21

UniprotID refers to protein identifiers on Uniprot.

Protein abbreviation is the abbreviation for each altered protein.

Log2 refers to the log2 fold change in PFOA exposed relative to vehicle control-treated lean mice.

A total of 129 proteins were altered in their level in the obese mice exposed to PFOA (*p *<* *.05). Eighty-eight proteins were increased and 41 were decreased (Figure 5B). The biological or molecular functions of 18 (12 increased and 6 decreased) proteins are involved in DNA damage and repair (Table 3), and 11 proteins (6 elevated and 5 reduced) in reproduction (Table 4).

**Table 3.** Ovarian Proteins Involved in DNA Damage Sensing and Repair Altered in PFOA-Exposed Relative to Vehicle Control-Treated Obese Mice

UniProtID	Protein Name	Protein Abbreviation	*p* Value	Log2(Fold Change)
P97431	Interferon regulatory factor 6	IRF6	.030	−1.01
O55128	Sin3A-associated protein 18	SAP18	.012	−0.84
Q8CH18	Cell division cycle and apoptosis regulator 1	CCAR1	.022	−0.79
Q9D2M8	Ubiquitin conjugating enzyme E2 V2	UBE2V2	.046	−0.77
P54728	RAD23 homolog B, nucleotide excision repair protein	RAD23B	.032	−0.58
Q9JKB3	Y-box-binding protein 3	YBX3	.016	−0.45
Q924A2	Capicua transcriptional repressor	CIC	.050	0.38
Q99MD9	Nuclear autoantigenic sperm protein	NASP	.047	0.43
P56546	C-terminal-binding protein 2	CTBP2	.021	0.46
P63085	Mitogen-activated protein kinase 1	MAPK1	.022	0.64
A2AWL7	MAX gene-associated protein	MGA	.011	0.76
Q8VCD5	Mediator complex subunit 17	MED17	.033	0.98
Q02780	Nuclear factor I A	NFIA	.036	1.04
P52479	Ubiquitin-specific peptidase 10	USP10	.028	1.12
Q9D1J3	SAP domain containing ribonucleoprotein	SARNP	.034	1.17
P52633	Signal transducer and activator of transcription 6	STAT6	.002	1.21
O08586	Phosphatase and tensin homolog	PTEN	.019	1.25
P42228	Signal transducer and activator of transcription 4	STAT4	.031	1.39

UniprotID refers to protein identifiers on Uniprot.

Protein abbreviation is the abbreviation for each altered protein.

Log2 refers to the log2 fold change in PFOA exposed relative to vehicle control-treated obese mice.

**Table 4.** Ovarian Proteins Involved in Reproduction Changed in PFOA-Exposed Relative to Vehicle Control-Exposed Obese Mice

UniProtID	Protein Name	Protein Abbreviation	*p* Value	Log2(Fold Change)
P29699	Alpha 2-HS-glycoprotein	AHSG	.009	−2.12
Q9CQK7	RWD domain containing 1	RWDD1	.005	−1.57
Q99L45	Eukaryotic translation initiation factor 2 subunit beta	EIF2S2	.043	−0.81
P54728	RAD23 homolog B, nucleotide excision repair protein	RAD23B	.032	−0.58
Q9JKB3	Y-box-binding protein 3	YBX3	.016	−0.45
Q08879	Fibulin 1	FBLN1	.010	0.49
P30416	FKBP prolyl isomerase 4	FKBP4	.017	0.49
P19001	Keratin 19	KRT19	.025	0.60
P63085	Mitogen-activated protein kinase 1	MAPK1	.022	0.64
P06745	Glucose-6-phosphate isomerase	GPI	.011	0.68

UniprotID refers to protein identifiers on Uniprot.

Protein abbreviation is the abbreviation for each altered protein.

Log2 refers to the log2 fold change in PFOA exposed relative to vehicle control-treated obese mice.

In the comparison between lean and obese controls, the abundance of 206 proteins were altered by obesity (*p *<* *.05). Of these, 93 were increased and 113 were decreased in their levels (Figure 5C). The biological or molecular functions of 39 (22 increased and 17 decreased) proteins are involved in DNA damage sensing and repair (Table 5), and 24 (10 increased and 14 decreased) proteins are involved in reproduction (Table 6).

**Table 5.** Ovarian DNA Damage Sensing and Repair Proteins Altered in Obese Relative to Lean Mice

UniProtID	Protein Name	Protein Abbreviation	*p* Value	Log2(Fold Change)
Q8CG72	ADP-ribosylserine hydrolase	ADPRS	.032	−3.745
P62878	Ring-box 1	RBX1	.042	−1.7921
O08586	Phosphatase and tensin homolog	PTEN	.0066	−1.7631
O88291	Zinc finger protein 326	ZNF326	.0064	−1.3845
Q8BZ21	Lysine acetyltransferase 6A	KAT6A	.0092	−1.2997
Q8CIG3	Lysine demethylase 1B	KDM1B	.0053	−1.1687
Q02248	Catenin beta 1	CTNNB1	.0092	−1.1661
P10639	Thioredoxin	TXN	.0052	−0.98829
P34884	Macrophage migration inhibitory factor	MIF	.012	−0.93639
Q80US4	Actin related protein 5	ACTR5	.046	−0.93475
P70288	Histone deacetylase 2	HDAC2	.0055	−0.92235
Q8CCK0	MacroH2A.2 histone	MACROH2A2	.048	−0.66778
Q9WV02	RNA-binding motif protein X-linked	RBMX	.016	−0.65389
Q8R081	Heterogeneous nuclear ribonucleoprotein L	HNRNPL	.011	−0.64919
Q8BM75	AT-rich interaction domain 5B	ARID5B	.0036	−0.55808
Q921I1	Transferrin	TF	.0033	−0.43368
O35381	Acidic nuclear phosphoprotein 32 family member A	ANP32A	.046	−0.40138
Q60973	RB-binding protein 7, chromatin remodeling factor	RBBP7	.032	0.27737
Q9CQV8	Tyrosine 3-monooxygenase/tryptophan 5-monooxygenase activation protein beta	YWHAB	.046	0.32484
Q61937	Nucleophosmin 1	NPM1	.0053	0.34355
P81117	Nucleobindin 2	NUCB2	.019	0.35221
Q9ERF3	SKI8 subunit of superkiller complex	SKIC8	.042	0.48708
P63101	Tyrosine 3-monooxygenase/tryptophan 5-monooxygenase activation protein zeta	YWHAZ	.043	0.51222
Q6PDG5	SWI/SNF-related, matrix-associated, actin-dependent regulator of chromatin subfamily c member 2	SMARCC2	.036	0.52058
Q8CH18	Cell division cycle and apoptosis regulator 1	CCAR1	.037	0.57973
O08749	Dihydrolipoamide dehydrogenase	DLD	.0014	0.58937
O55128	Sin3A-associated protein 18	SAP18	.022	0.59521
Q91YE6	Importin 9	IPO9	.024	0.59599
O88543	COP9 signalosome subunit 3	COPS3	.013	0.61813
Q5RJI5	BR serine/threonine kinase 1	BRSK1	.048	0.62241
O88700	BLM RecQ like helicase	BLM	.020	0.72346
P68510	Tyrosine 3-monooxygenase/tryptophan 5-monooxygenase activation protein eta	YWHAH	.013	0.75795
P61079	Ubiquitin conjugating enzyme E2 D3	UBE2D3	.013	0.79235
P42227	Signal transducer and activator of transcription 3	STAT3	.037	0.91826
P08775	RNA polymerase II subunit A	POLR2A	.013	0.97614
Q8CGB3	Uveal autoantigen with coiled-coil domains and ankyrin repeats	UACA	.013	1.3975
Q8CIG8	Protein arginine methyltransferase 5	PRMT5	.043	1.4058
Q02395	Metal response element-binding transcription factor 2	MTF2	.0057	1.434
Q2VPU4	MLX interacting protein	MLXIP	.015	1.8047

UniprotID refers to protein identifiers on Uniprot.

Protein abbreviation is the abbreviation for each altered protein.

Log2 refers to the log2 fold change in obese relative to lean mice.

**Table 6.** Ovarian Reproduction Proteins Altered by Obesity

UniProtID	Protein Name	Protein Abbreviation	*p* Value	Log2(Fold Change)
Q06770	Serpin family A member 6	SERPINA6	.0057	−3.6512
O08586	Phosphatase and tensin homolog	PTEN	.0066	−1.7631
P26361	CF transmembrane conductance regulator	CFTR	.041	−1.4367
Q02248	Catenin beta 1	CTNNB1	.0092	−1.1661
O70167	Phosphatidylinositol-4-phosphate 3-kinase catalytic subunit type 2 gamma	PIK3C2G	.042	−0.91511
Q6NZC7	SEC23 interacting protein	SEC23IP	.042	−0.57556
Q8BM75	AT-rich interaction domain 5B	ARID5B	.0036	−0.55808
Q14AT2	Testis expressed 11	TEX11	.0036	−0.55572
P48036	Annexin A5	ANXA5	.036	−0.51797
P30416	FKBP prolyl isomerase 4	FKBP4	.026	−0.44588
P54869	3-hydroxy-3-methylglutaryl-CoA synthase 2	HMGCS2	.040	−0.37046
O88844	Isocitrate dehydrogenase (NADP(+)) 1	IDH1	.018	−0.33729
P06745	Glucose-6-phosphate isomerase	GPI	.025	−0.31511
P24815	Hydroxy-delta-5-steroid dehydrogenase, 3 beta- and steroid delta-isomerase 1	HSD3B1	.040	−0.26552
Q60973	RB-binding protein 7, chromatin remodeling factor	RBBP7	.032	0.27737
P08228	Superoxide dismutase 1	SOD1	.044	0.2967
P81117	Nucleobindin 2	NUCB2	.015	0.35221
O08749	Dihydrolipoamide dehydrogenase	DLD	.0014	0.58937
P29699	Alpha 2-HS glycoprotein	AHSG	.0059	0.68981
Q99LD9	Eukaryotic translation initiation factor 2B subunit beta	EIF2B2	.016	0.69071
P42227	Signal transducer and activator of transcription 3	STAT3	.037	0.91826
Q6R891	Protein phosphatase 1 regulatory subunit 9B	PPP1R9B	.039	1.0235
P16627	Heat shock protein family A (Hsp70) member 1 like	HSPA1L	.042	1.1903
B2RV46	Spermatogenesis associated 6 like	SPATA6L	.0037	2.1449

UniprotID refers to protein identifiers on Uniprot.

Protein abbreviation is the abbreviation for each altered protein.

Log2 refers to the log2 fold change in obese relative to lean mice.

In both lean and obese mice, 4 proteins involved in processes relevant to both DNA damage sensing and reproduction that were changed in their level by PFOA exposure (log2(fold-change)) were: calcium homeostasis endoplasmic reticulum protein (CHERP) (lean: 0.78-fold increase; obese: 0.77-fold increase), hemopexin (HPX) (lean: 0.53-fold increase; obese: 1.1-fold increase), opioid growth factor receptor (OGFR) (lean: −1.0-fold decrease; obese: −0.45-fold decrease), and alpha 2-HS glycoprotein (AHSG) (lean: −0.82-fold decrease; obese: −2.1-fold decrease) (Figure 5D).

**Figure 6.**
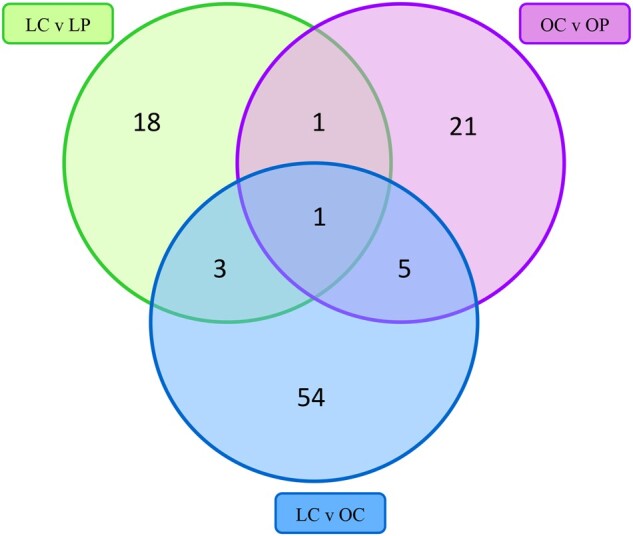
Proteins involved in DNA damage sensing and repair and reproduction that are in common or unique between treatments. The Venn diagram presents the number of ovarian proteins identified as being unique to treatment or altered in common by treatments involved in DNA damage sensing and repair and reproduction. The number of proteins in the green circle indicates the number of proteins identified in the comparison between LC and LP groups; the purple circle indicates the number of proteins identified in the comparison between OC and OP groups; and the blue circle indicates the number of proteins identified in the comparison between LC and OC groups. Overlapping areas of the circles illustrate the number of proteins that were altered by 2 or more groups.

Of these proteins, 23, 28, and 63 proteins were identified that were unique to LC versus LP, OC versus OP, and LC versus OC, respectively. In addition, 1 protein was shared between LC versus LP and OC versus OP groups, 3 proteins were shared between LC versus LP and LC versus OC, 5 proteins were shared between OC versus OP and LC versus OC groups, and 1 protein was shared between the 3 comparisons (Figure 6).

## DISCUSSION

Per- and polyfluoroalkylated substances have been used in industrial and commercial products since the 1940s (Lindstrom *et al.*, 2011) because of their ability to repel water and oil (Bell *et al.*, 2021). The U.S. Environmental Protection Agency (EPA) has not established maximum contaminant level of PFOA for drinking water (USEPA, 2017). However, newly issued advice from the EPA established drinking water health advisories of 0.004 ppt for PFOA and 0.02 ppt for PFOS. In the European Union, the Scientific Panel on Contaminants in the Food Chain (CONTAM) of the European Food Safety Authority (EFSA), established the Tolerable Daily Intake for PFOA of 1.5 µg/kg body weight per day (EFSA, 2008). From 1999 to 2016, the National Health and Nutrition Examination Survey (NHANES) demonstrated PFOA concentrations in the serum of the general U.S. population (Centers for Disease Control and Prevention, 2021a,b) and a study from the Netherlands estimated long-term intake median values for PFOA as 0.2 ng/kg/day and PFOS as 0.3 ng/kg/day (Noorlander *et al.*, 2011). An arithmetic mean serum concentration of 691 ng/ml (range 72–5100 ng/ml) PFOA can occur through occupational exposure (Olsen *et al.*, 2007). In humans, the PFOA half-life of elimination is approximately 2.7 and 3.8 years (Li *et al.*, 2018; Olsen *et al.*, 2007). In rats, the half-life is approximately 2–4 h in females (Vanden Heuvel *et al.*, 1991), and from 4 to 9 days in males (EFSA, 2008), attributed to biological sex differences in renal excretion (Vanden Heuvel *et al.*, 1991). Similarly, PFOA half-life is 15 days and 21 days in female and male mice, respectively (Lou *et al.*, 2009).

The main routes of human exposure to PFOA are ingestion of contaminated food (both from animal exposure and food packaging) and drinking water followed by indoor air and dust inhalation, and house dust transfer from hand to mouth (Trudel *et al.*, 2008). After exposure, PFOA is readily absorbed (EFSA, 2008) and PFAS are not considered to be biotransformed *in vivo* (ATSDR, 2021; Kemper and Nabb, 2005; Vanden Heuvel *et al.*, 1991) before being eliminated mostly through urine, and bile (Vanden Heuvel *et al.*, 1991). Accumulation of PFAS compounds occurs in blood, liver, kidney, testicles, brain (Jensen and Leffers, 2008), and ovaries (Vanden Heuvel *et al.*, 1991). Human follicular fluid can contain PFOA (Heffernan *et al.*, 2018) thus, PFAS could be a potential exposure for the oocyte (Ding *et al.*, 2020). Although there are geographical as well as human ethnicity (Park *et al.*, 2019) influences for PFAS exposure, the daily cumulative PFOA exposure remains unclear. To determine an ovarian impact of PFOA exposure, a relevant oral route of exposure and a dose of 2.5 mg/kg PFOA was examined which is intermediate for the LOAEL noted for PFOA in the testis (5 mg/kg; Li *et al.*, 2011) and the liver (1 mg/kg; Loveless *et al.*, 2008). A PFOA dose of 5 mg/kg was recently determined to cause ovarian follicle loss in mice (Yang *et al.*, 2022), though the modes of action outside of steroidogenesis mRNA gene abundance were not examined.

At 7 weeks of age, lean and obese mice were orally exposed to 2.5 mg/kg body weight of PFOA for 15 days. A hyperphagia-induced model of obesity with a mutation of the agouti gene in the hypothalamus, which increases agouti expression decreasing melanocyte-stimulating hormone resulting in overeating (Duhl *et al.*, 1994; Klebig *et al.*, 1995; Lu *et al.*, 1994; Michaud *et al.*, 1993, 1994) was utilized. The lean counterparts are of the same genetic background and eat the same diet composition, but they consume less calories. This obesity model has altered circulating insulin (Yang *et al.*, 2012), alterations to steroidogenesis (Nteeba *et al.*, 2014b), decreased number of primordial follicles from 12 weeks of age onwards (Ganesan *et al.*, 2017), and a diminished response to environmental toxicants (Ganesan *et al.*, 2014, 2017; González-Alvarez *et al.*, 2021). The obese mice were approximately 24% heavier than the lean mice at the end of dosing and this age was chosen to eliminate differences in ovarian follicle composition between the lean and obese mice at the start of PFOA exposure which has been noted from 12 weeks of age onward in this obese model (Nteeba *et al.*, 2014b). It is recognized that this is likely at the moderate level of weight gain in women and greater effects may have been identified if a greater weight attainment was achieved, however, consideration of removing the confounding impact of obesity-induced follicle loss was important.

Conflicting results have been reported in animal studies and epidemiological studies that studied the relationship between exposure to PFOA and body weight. In humans, inverse associations between maternal blood and umbilical cord serum PFOA levels with birth weight and size are reported in some studies (Apelberg *et al.*, 2007; Bach *et al.*, 2015; Fei *et al.*, 2007) but not others (Andersen *et al.*, 2013; Høyer *et al.*, 2015; Monroy *et al.*, 2008; Nolan *et al.*, 2009). Prenatal PFOA plasma levels have been associated with increased BMI in girls but not boys (Mora *et al.*, 2017). Increased body weight due to PFOA exposure has been reported in rats (Du *et al.*, 2019) but the opposite in PFOA-exposed mice (Wolf *et al.*, 2007; Yang *et al.*, 2009). In this study, the agouti overexpressing mice were heavier than their lean counterparts due to experimental design but there was no impact of PFOA exposure on body weight in either the lean or obese mice indicating lack of overt toxicity. In agreement with our findings, female BALB/c mice (6–8 weeks of age) had no effect of PFOA on body weight (Fairley *et al.*, 2007), though a different route of exposure and dosage was used (Fairley *et al.*, 2007). Most studies documenting PFOA-induced differences in body weight have used higher concentrations than in this study. Other potential reasons for differential impacts of PFOA on body weight include different strains of mice, differences between species, as well as the dosing duration and animal stage of development.

Food intake and the weight of heart, spleen, kidney, and uterus were not affected by PFOA exposure or obesity. Decreased uterine weight has been reported in PFOA exposed (1, 5, and 10 mg/kg) BALB/c and C57BL/6 mice (Yang *et al.*, 2009) albeit in a dose- and strain-dependent manner (Yang *et al.*, 2009). Lower and acute PFOA exposure (0.01 mg/kg/day) increased uterine weight (Dixon *et al.*, 2012), supporting influence of the dosing paradigm. Dermal PFOA exposures decreased spleen weight (Fairley *et al.*, 2007) and adverse impacts on spleen weight were also noted in male C57BL/6 mice (Yang *et al.*, 2002). As with body weight, potential explanations for differences between the current study and others include animal model, dose, and duration of PFOA exposure.

Ovarian weight was increased in neonatal female rats (PND 1–5), and juvenile rats (PND 26–30) exposed to 1 mg/kg and 10 mg/kg of PFOA, respectively, for 5 days (Du *et al.*, 2019). There was no effect on ovarian weight in pregnant female Kunming mice that received gestational exposure to 2.5, 5, or 10 mg/kg/day of PFOA (Chen *et al.*, 2017). CD-1 female mice at 30 days of age exposed to 5, 10, and 20 mg/kg of PFOA for 10 days did not have altered ovarian and uterine weight (Yang *et al.*, 2022). In the current study, ovarian weight was decreased in the lean but not obese mice exposed to PFOA. Lack of an effect of PFOA exposure on ovarian weight in the obese mice illustrates differences in the PFOA response in the obese relative to lean mouse ovary. The decrease in ovary weight might be explained by follicle loss caused by PFOA exposure, because PFOA was demonstrated to reduce the number of primordial and growing follicles (Du *et al.*, 2019; Yang *et al.*, 2022) as well as corpora lutea (Chen *et al.*, 2017; Du *et al.*, 2019). Furthermore, PFOA and PFOS can disrupt gap junction communication between granulosa cells and oocytes in mice and swine (Dominguez *et al.*, 2016; Lopez-Arellano *et al.*, 2019) potentially resulting in oocyte death and follicle loss. These differences might be also attributed to the dose level, dosing duration, mouse strain, and the developmental status.

Per- and poly-fluorinated compounds are potential endocrine disrupting chemicals (Jensen and Leffers, 2008). In this study, there was no difference between the lean and obese mouse response to PFOA exposure on E_2_ serum levels when samples lower than the level of detection of the assay were included, however omission of those below assay detection limit samples indicated that circulating E_2_ was increased in obese but not lean mice and is an avenue for further exploration. Perhaps due to blood being collected during diestrus, several samples were below the detectable range of the E_2_ assay resulting in a smaller sample size in the lean group compared with the obese group. In the future, ovaries at the estrus cycle stage could be collected for a more appropriate quantification of the effects of E_2_. Serum P_4_ concentration was not affected by PFOA exposure. A study in reproductive aged women discounted an association between PFOA exposure and E_2_ serum concentrations (Knox *et al.*, 2011). An association between E_2_ and testosterone (T_4_) with serum PFOA level was demonstrated in men, but not in women (Steenland *et al.*, 2010). No association between PFOA serum level and E_2_ levels in Taiwanese girls (Zhou *et al.*, 2016) or between serum PFOA, E_2_, and P_4_ in women has been reported (Barrett *et al.*, 2015). In animal models, serum E_2_ was increased on GD7 in pregnant female Kunming mice exposed to 10 mg/kg/day of PFOA (Chen *et al.*, 2017). Additionally, on GD13, serum P_4_ was decreased in mice exposed to 5 and 10 mg/kg/day of PFOA (Chen *et al.*, 2017). Neonatal exposure (PND 1–5) to 0.1 and 1 mg/kg/day PFOA for 5 days increased serum E_2_ in female rats (Du *et al.*, 2019). Young female C57BL/6 mice exposed to 5 mg/kg of PFOA for 5 days per week for 4 weeks (Zhao *et al.*, 2010) had no observed alteration in serum E_2_, but P_4_ was increased during the estrus and proestrus stages (Zhao *et al.*, 2010). In mice exposed to 1, 5, 10, or 20 mg/kg PFOA, there was no impact on E_2_, but P_4_ was decreased at 5 mg/kg exposure (Yang *et al.*, 2022). Secretion of P_4_ was not altered by 0.012–24 µM PFOA exposure in cultured porcine theca cells (Chaparro-Ortega *et al.*, 2018). However, in granulosa cells, P_4_ and E_2_ secretion were decreased at 0.12 µM and 0.012 µM, respectively, indicating a concentration dependence (Chaparro-Ortega *et al.*, 2018). Thus, in this study, an endocrine disrupting impact of PFOA cannot be discounted and is worthy of future investigation.

The time spent in proestrus, estrus, metestrus, and diestrus stages were not affected by PFOA exposure, obesity, nor was there any additive effect of obesity and PFOA. In women, irregular and longer menstrual cycles have been associated with higher levels of serum PFOA (Fei *et al.*, 2009; Lyngsø *et al.*, 2014; Zhou *et al.*, 2017) although other studies reported no association between menstrual cycle length and serum PFOA level (Lum *et al.*, 2017). Neonatal exposure of rats to 0.1 and 1 mg/kg/day PFOA for 5 days affected post-pubertal estrous cyclicity (Du *et al.*, 2019), but exposure to 10 mg/kg/day of PFOA caused temporary post-pubertal acyclicity (Du *et al.*, 2019). Juvenile mouse exposure (PND 26–30) to 1 mg/kg/day PFOA also resulted in irregular cyclicity (Du *et al.*, 2019). Lack of a phenotypic impact of estrous cyclicity could translate to an insidious impact of PFOA exposure that may not be apparent to an affected female.

Based upon the previous studies of PFOA-induced follicle loss at similar exposure levels (Yang *et al.*, 2022), this study focused instead on proteomic impacts within the ovary. Altered ovarian abundance of proteins involved in DNA damage sensing and repair and reproduction were examined in ovaries of lean and obese mice exposed to PFOA. Several DDR proteins were altered by PFOA exposure in ovaries from lean mice. A nuclear helicase protein (Li *et al.*, 2016), HELZ interacts with and stimulates the peroxisome proliferator-activated receptor α (PPARα); (Surapureddi *et al.*, 2002) and is involved in DNA repair, gene transcription, and RNA processing (Katano-Toki *et al.*, 2013). In the ovary, PPAR α, β/δ, and γ are detectable in granulosa, theca, and stromal cells and in the corpus luteum (Ding *et al.*, 2020). Members of the PPAR family are involved in oogenesis, folliculogenesis, and ovulation (Ding *et al.*, 2020). Lean PFOA mice had decreased levels of ovarian HELZ2 which could disrupt PPAR signaling.

Linker histone (H1) family members, including the subtype H1.4 linker histone, cluster member (H1-4) stabilizes chromatin and is essential for chromatin fiber maintenance and assembly (Bednar *et al.*, 2017; Hergeth and Schneider, 2015). Ovarian H1-4 is increased by PFOA exposure in lean mice and alterations in levels of H1 family members can upregulate or downregulate specific genes (Hergeth and Schneider, 2015). The level of H1 phosphorylation is indicative of cellular DNA damage level (Hergeth and Schneider, 2015) and expression of H1 subtypes are proposed as biomarkers for ovarian cancer (Medrzycki *et al.*, 2012). The interaction between linker histones H1 with chromatin is regulated by Prothymosin alpha (PTMA; Gomez-Marquez and Rodríguez, 1998; Karetsou *et al.*, 1998, 2004). Increased PTMA is observed in breast (Tsitsiloni *et al.*, 1993), and prostate cancer (Suzuki *et al.*, 2006) as well as other types of aggressive cancer (Tsitsiloni *et al.*, 1993). Decreased PTMA slows intranuclear linker histone exchange between chromatin sites (George and Brown, 2010) altering cell proliferation and replication and in lean mice exposed to PFOA, ovarian PTMA was decreased potentially leading to ovotoxicity.

The switch/sucrose non-fermenting (SWI/SNF) complex includes SWI/SNF related, matrix associated, actin-dependent regulator of chromatin, subfamily a, member 4 (SMARCA4) (Hodges *et al.*, 2016), and involved in chromatin remodeling to regulate DNA replication and repair (Hodges *et al.*, 2016). Promotion of HR repair is facilitated by SMARCA4 (Kurashima *et al.*, 2020) and in the ovary, alterations in SMARC4 have been associated with ovarian carcinomas (Agaimy *et al.*, 2015; Moes-Sosnowska *et al.*, 2015). Ovarian abundance of SMARCA4 is decreased by PFOA exposure in lean mice, potentially altering the capacity of the ovary to respond to DNA damage.

Several proteins associated with DDR were also noted to be altered by PFOA exposure in obese mice including MGA which is involved in meiosis in embryonic and germline stem cells (Suzuki *et al.*, 2016). An *Mga* mRNA variant has been found in meiotic germ cells and round spermatids (Kitamura *et al.*, 2021). During oogenesis, oocytes that arise from primordial germ cells become arrested in meiosis prophase I, and meiosis resumes in the oocyte at the time of ovulation (Bahr and Milich, 2013; Hirshfield, 1991). After meiosis I is completed, meiosis II starts; however, this is also arrested in the second meiotic metaphase until fertilization occurs (Bahr and Milich, 2013; Hirshfield, 1991). In obese mice exposed to PFOA, MGA ovarian abundance was increased potentially leading to negative meiotic effects.

The mediator complex which includes MED17 (Kikuchi *et al.*, 2015) has essential roles in transcription factor regulation (Malik and Roeder, 2010). Transcription is positively regulated by MED17, including the NER pathway, functioning as a switch between transcription and DNA repair (Kikuchi *et al.*, 2015). In obese mice, PFOA exposure increased ovarian abundance of MED17, potentially indicating sensing of DNA damage and activation of NER pathways.

RAD23 homolog B, NER protein (RAD23B) is part of the xeroderma pigmentosum protein C (XPC)-RAD23-Centrin 2 (CEN2) complex, which senses and recruits repair factors to sites with DNA lesions in the NER pathway (GG-NER) (Bergink *et al.*, 2012; Ng *et al.*, 2003). Expression of circular RNA *Rad23b* was high in ovarian cancer tissues (Yu *et al.*, 2021). Relevant to reproduction, RAD23B has been associated with the proper embryonic development, placental formation, and spermatogenesis (Ng *et al.*, 2002). In the obese mice, ovarian protein abundance of RAD23B was increased by PFOA exposure, which might indicate that there is an increase in DNA damage that might lead to ovarian cancer or other reproductive effects.

AHSG is a glycoprotein that inhibits insulin receptor tyrosine kinase and is associated with insulin resistance, diabetes type 2, and metabolic syndrome (Dabrowska *et al.*, 2015). Increased plasma AHSG was associated with inactive ovaries during early lactation in dairy cows (Zhao *et al.*, 2019). In this study, exposure to PFOA decreased ovarian abundance of AHSG in both lean and obese mice. Decreased AHSG might increase insulin signaling activating PI3K signaling, which regulates both viability (Brown *et al.*, 2010) and activation of primordial follicles (Jagarlamudi *et al.*, 2009; Liu *et al.*, 2006; Reddy *et al.*, 2005). Induction of low-grade inflammation can be induced by AHSG (Dabrowska *et al.*, 2015), and PFAS levels have been linked with inflammation (Salihovic *et al.*, 2020). Thus, there is potentially increased ovarian inflammation due to PFOA exposure in the lean and obese mice. The action of PI3K is antagonized by phosphatase and tensin homolog deleted on chromosome 10 (PTEN) (Cantley, 2002). In the ovary, PTEN (oocyte-specific) null female mice undergo global primordial follicle activation (Jagarlamudi *et al.*, 2009) and PFOA exposure increased PTEN abundance in obese mice. Thus, altered PI3K via PTEN caused by PFOA exposure could alter primordial follicle viability contributing to primordial follicle loss as documented recently (Yang *et al.*, 2022).

In both lean and obese mice, PFOA exposure increased the abundance of CHERP and HPX but decreased AHSG and OGFR. Increased CHEPR could indicate endoplasmic reticulum stress due to PFOA exposure and within the ovary, this is documented to have roles in apoptosis (Huang *et al.*, 2017) and reproductive dysfunction (Guzel *et al.*, 2017). Increased ovarian levels of HPX was caused by PFOA exposure in both lean and obese mice, and although there is little documented regarding functions of ovarian HPX, it is an acute-phase protein that is present in follicular fluid (Angelucci *et al.*, 2006) and higher levels of HPX precursor were identified in the follicular fluid of older compared with young women (Hashemitabar *et al.*, 2014), potentially indicating a role in ovarian aging. Exposure to PFOA decreased the abundance of the OGFR in both lean and obese mice. The opioid growth factor (OGF) and the OGFR axis have decreased abundance in ovarian cancer (Fanning *et al.*, 2012). The OGF delays the cell cycle (Fanning *et al.*, 2012), potentially facilitating DNA repair, and also has roles in ovarian steroidogenesis (Kaminski *et al.*, 2004). Exposure to PFOA has been determined to negatively impact steroidogenesis, with the OGF-OGFR being a potential mediator of this effect.

In summary, PFOA exposure differentially altered several aspects of ovarian biology between lean and obese mice, indicating an influence of body composition on PFOA-induced reproductive toxicity as we have noted with other chemicals (Clark *et al.*, 2020; Ganesan *et al.*, 2014, 2017; González-Alvarez *et al.*, 2021; Nteeba *et al.*, 2014a). Exposure to PFOA did not impact feed intake, body weight, or estrous cyclicity in either the lean or obese mice. In lean mice, ovarian weight was lower than control-treated mice with lack of this impact in the obese mice. In obese mice, PFOA exposure increased E_2_ level, albeit from a lower control level than in the lean mice. The DNA damage sensing and repair as well as reproductive protein response to PFOA exposure also differed between lean and obese mice. Taken together, these data help to provide an insight in possible mechanisms in which PFOA may induce ovarian toxicity and that the difference in the physiological status causes differences in the ovotoxicity induced by PFOA. It is recognized that enhanced geographical and ingestion PFOA/PFAS surveillance is needed to establish human intake exposures in both nonoccupational and occupational situations to better design studies upon which to base reproductive risk assessment.

## FUNDING

Bailey Career Development Grant from Iowa State University to A.F.K.

## DECLARATION OF CONFLICTING INTERESTS

The authors declared no potential conflicts of interest with respect to the research, authorship, and/or publication of this article. The authors do not have any conflicts of interest.
